# The Treatment of Stricture of the Anterior Urethra

**Published:** 1903-06

**Authors:** W. L. Champion

**Affiliations:** Atlanta, Ga.


### THE TREATMENT OF STRICTURE OF THE ANTERIOR URETHRA.*

BY W. L. CHAMPION, M.D.,

ATLANTA, GA.

The object of this short paper is to express my individual opinion as to the best means of treating stricture of the anterior or pendulous urethra. While the suggestions offered follow those of Otis and Lydston, they were gained from personal experience, and are presented to stimulate better work in urethral surgery. It

* Read at meeting Medical Association of Georgia, Columbus, April, 1903.

might be well to state that with few exceptions urethral stricture is due to gonorrhea; "that a chronic gonorrhea or gleet usually means urethral stricture," and that the urethral discharge from ninety-five per cent. of the patients we treat—whether acute or chronic—will show the gonococcus present. I examined the discharge from the urethras of 368 consecutive cases in private practice with the results stated above, regardless of the length of time the disease had existed.

Considering the above facts, that chronic gonorrhea usually means urethral stricture; that in ninety-five per cent. of the cases with a purulent or muco-purulent discharge the gonococcus is present, we can readily see the importance of carefully examining the urethra for stricture, and, if present, to use the best means of removing it.

In regard to examining the urethra, it should be remembered the sound is not an instrument for diagnosis and should not be used for that purpose. The bulbous bougie, or urethrometer, is the instrument to determine whether the urethra is strictured. A full size sound may readily pass into the bladder of a patient with a stricture of large caliber without its presence being known, when the bulbous bougie, or urethrometer, will demonstrate a fibrous contraction. An abnormal narrowing of the urethra is a mechanical obstruction to the flow of urine, and causes the retention of a certain amount of urine in the urethra. This condition naturally produces a chronic state of inflammation which finally extends to the prostate gland, seminal vesicles and other adjacent structures.

As to the various methods of treating urethral stricture—by divulsion, electrolysis, dilatation and cutting—the only cases I have gotten satisfactory results from followed the latter operation. After using electrolysis in fifty-two cases of stricture I did not get the results claimed by others. Dilatation is the proper method of treatment in strictures of recent formation—that is, inflammatory thickening, and not fibrous contractions. To get permanent relief from organic stricture is to completely sever the fibrous bands or tissue with the knife, and by the use of sounds make the cut or strictured portion of the canal heal to the size of the normal urethra. The failures recorded after internal urethrotomy are due to the fact of the strictured tissues not being completely severed, or failure to introduce the sound at proper intervals after the operation. The fact that we frequently find more than one stricture should not be overlooked, and the operator should be careful not to leave a single fibrous band uncut, as the success of the operation may hinge on this point. As a rule strictures in the penile urethra are located within the first three inches, but it is frequently the case that contractions are deeper that require cutting. I do not consider it safe to do an internal urethrotomy without perineal drainage deeper than five inches. It is a fact that if a strictured urethra is properly cut, and the after-treatment carried out (which is as important as the cutting), the urethra can be examined years afterward and found free of contractions. The operation of internal urethrotomy should not be done in the surgeon's office. The patient should be required to remain in bed three or four days. The sound can be passed on the third day, and the patient allowed to return to his business on the fourth. With a two per cent. solution of cocain the operation can be done with very little pain. Adrenalin used after the operation will control the hemorrhage. It is advisable to pass the sound every four or five days until the hemorrhage ceases, then every ten days for three or four months. In a brief paper of this character, the various kinds of urethral stricture—their location, causation and symptomatology—cannot be considered, nor the fact that nearly all strictured urethras are infected urethras; but the point I wish to emphasize is that internal urethrotomy is the proper method of curing fibrous stricture of the anterior urethra.

REFERENCE: Stricture of the Urethra, by G. Frank Lydston, M.D.

---

GRADUATES of an institution of osteopathy, requiring four courses of five months each, are now eligible to practice osteopathic medicine (?) and osteopathic surgery (?) in New Mexico, but are precluded from using drugs or performing major surgical operations under penalty of committing a misdemeanor. This is the outcome of a five-year conflict between the New Mexico Medical Society and the osteopaths.—*New York Medical Journal.*

